# Supplementary material for: Arg18 Substitutions Reveal the Capacity of the HIV-1 Capsid Protein for Non-Fullerene Assembly
Source: Viruses. 2024 Jun 27;16(7):1038. doi: 10.3390/v16071038 (PMC11281672; doi:10.3390/v16071038)
Supplement: Supplementary file 1 [file viruses-16-01038-s001.zip › viruses-2895639-supplementary.pdf]

Supplementary Information

Arg18 substitutions reveal the capacity of the HIV-1 capsid protein for non-fullerene assembly

Randall T. Schirra[1], Nayara F. B. dos Santos[1,2], Barbie K. Ganser-Pornillos[1,2,*], Owen Pornillos[1,2,*]

[1]Department of Molecular Physiology and Biological Physics, University of Virginia, Charlottesville, VA 22903, USA

[2]Department of Biochemistry, University of Utah, Salt Lake City, UT 84112, USA

*Correspondence: owen@biochem.utah.edu, barbie.pornillos@cores.utah.edu

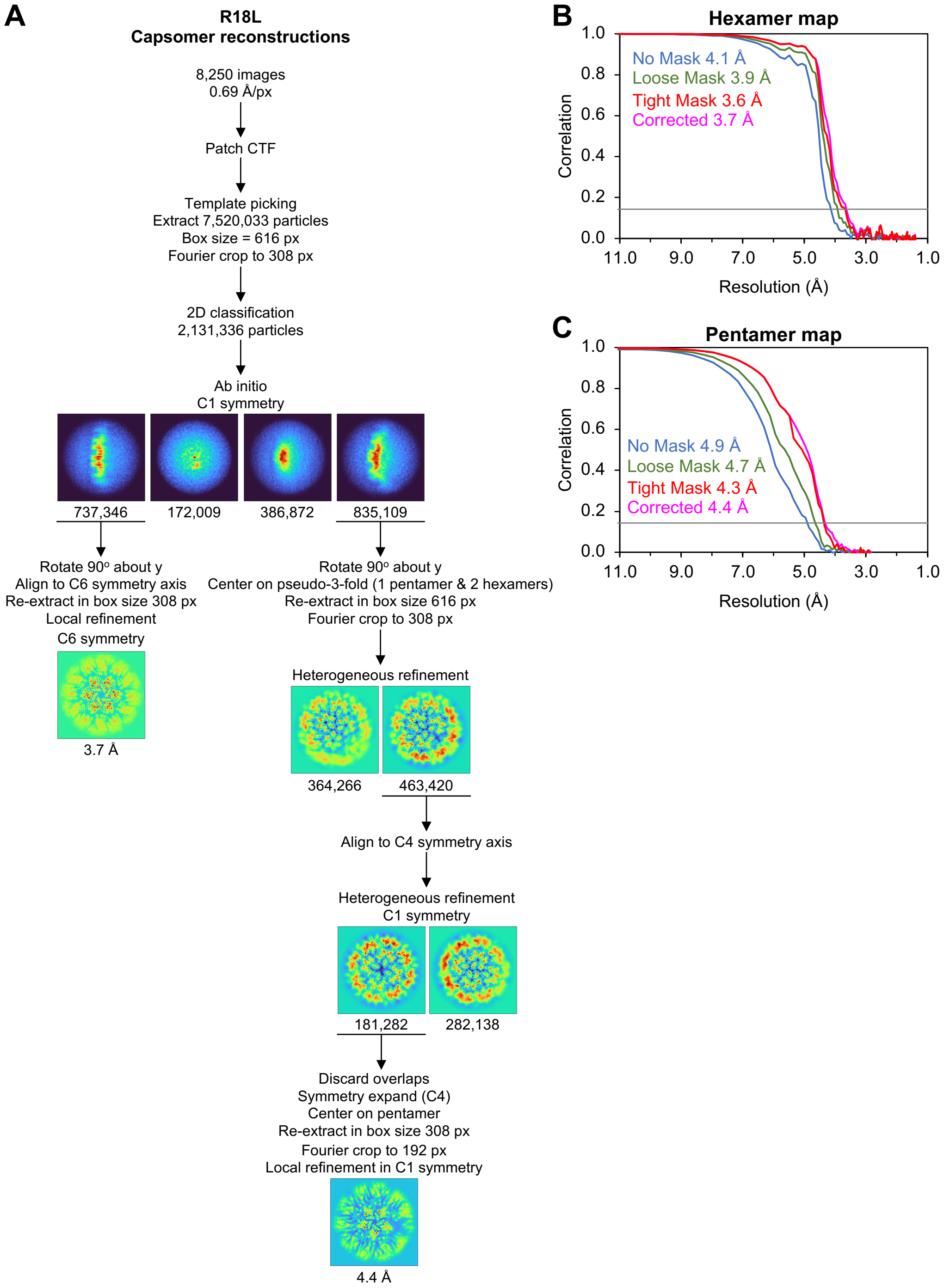

Supplementary Figure 1. Focused reconstructions of R18L capsomers. (**A**) CryoEM workflow. (**B,C**) Fourier shell correlation curves for the indicated maps.

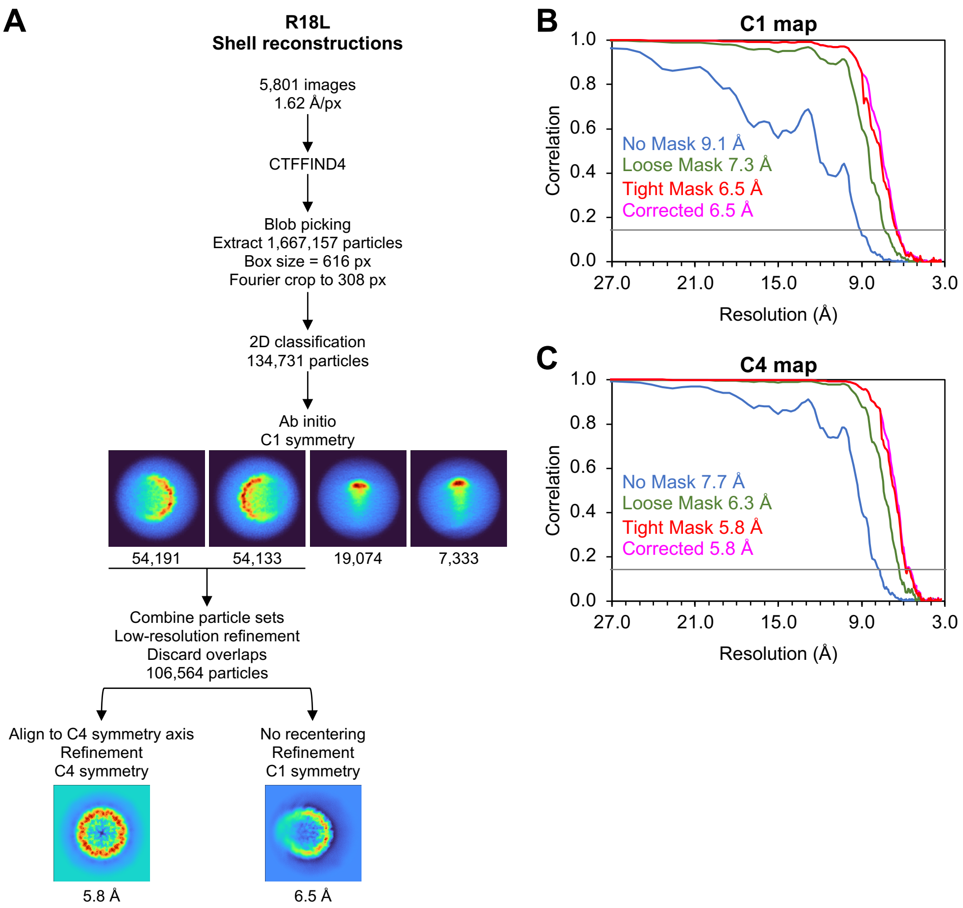

Supplementary Figure 2. Shell reconstructions of R18L CLPs. (**A**) CryoEM workflow. (**B,C**) Fourier shell correlation curves for the indicated maps.

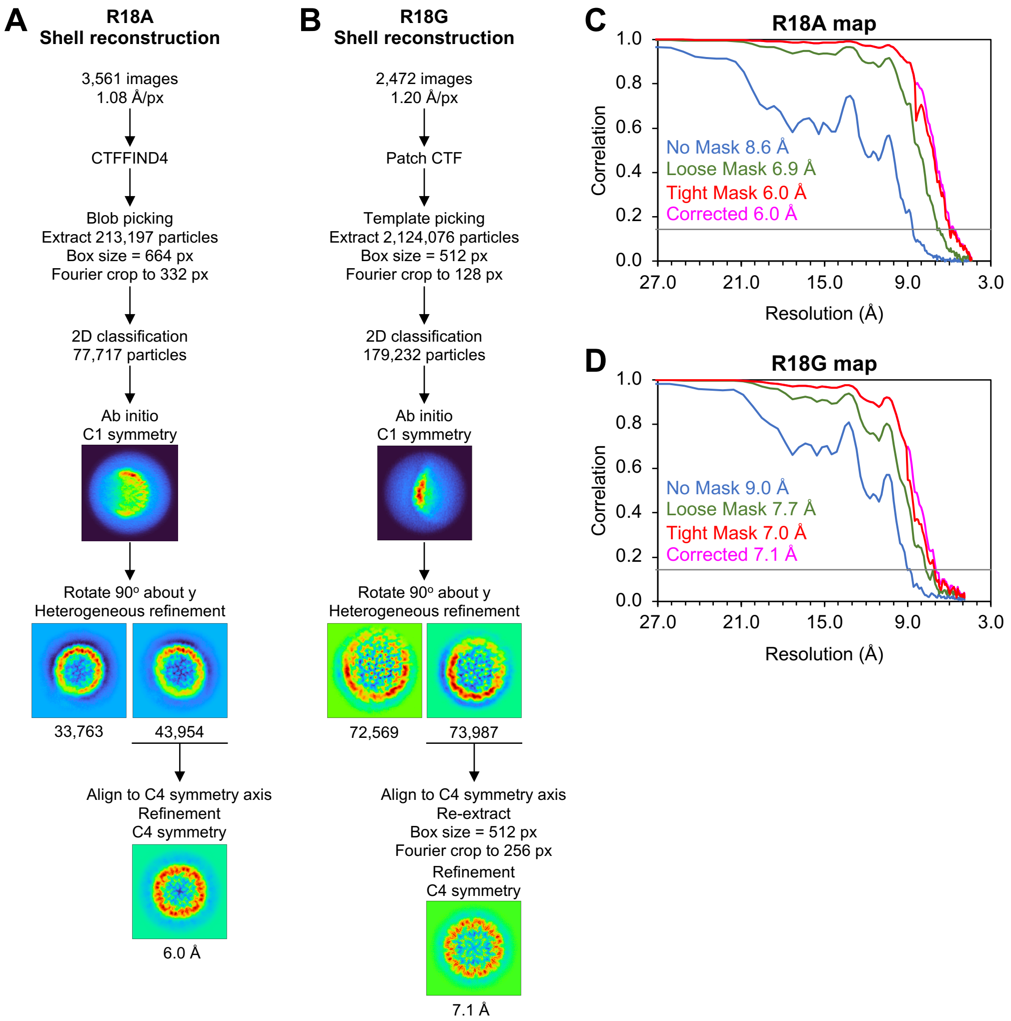

Supplementary Figure 3. Shell reconstructions of R18A and R18G CLPs. (**A,B**) CryoEM workflows. (**C,D**) Fourier shell correlation curves for the indicated maps.

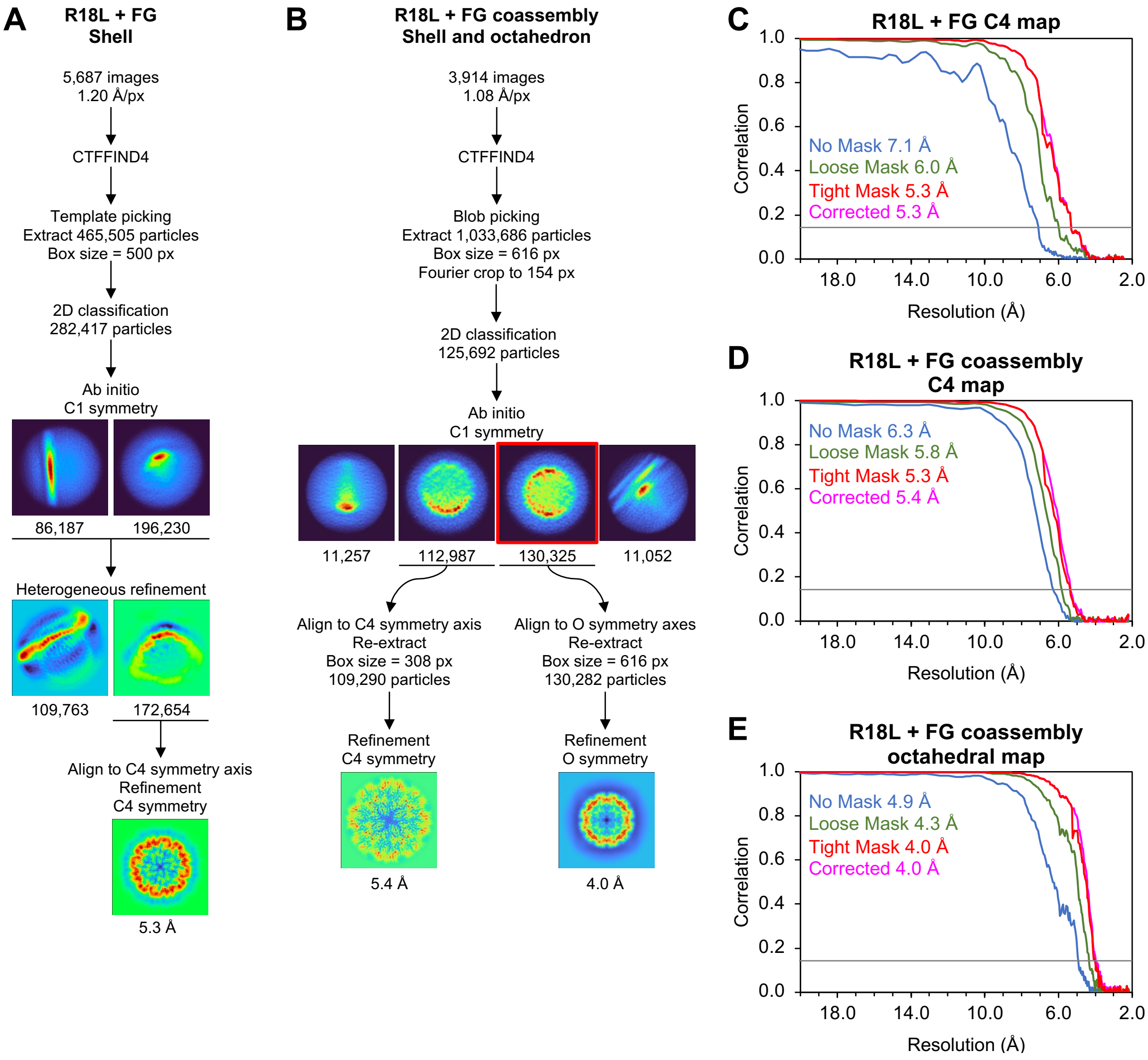

Supplementary Figure 4. Reconstructions of R18L CLPs in complex with CPSF6-FG peptide. (**A,B**) CryoEM workflows for the indicated samples. (**C-E**) Fourier shell correlation curves for the indicated maps.

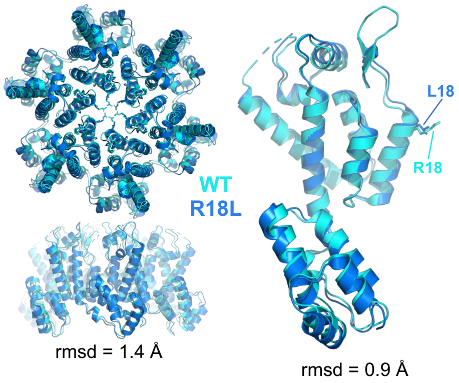

Supplementary Figure 5. Comparison of the R18L and WT hexamers. Structures of WT (cyan, PDB 8ckv [13]) and R18L (blue, from this study) were superimposed either as entire hexamer units (left), or as single CA subunits (right). Root mean square deviations (rmsd) were calculated for all equivalent Cα atoms.

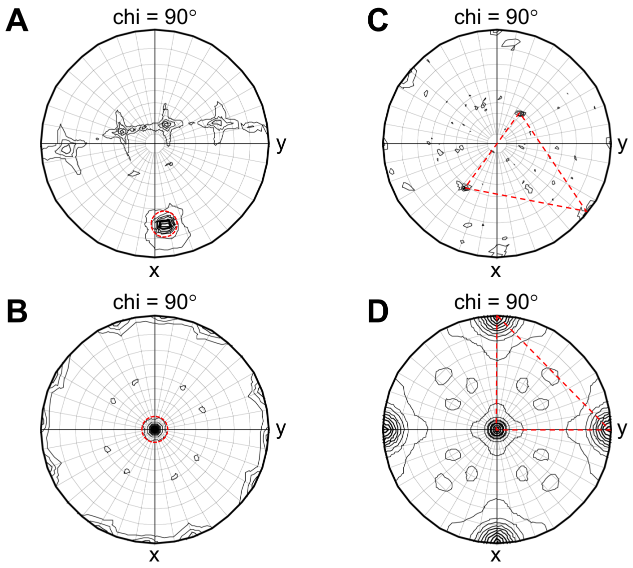

Supplementary Figure 6. Stereographic projections of self-rotation functions. (**A**) Final refined map of R18L tetramer-of-pentamers without symmetry imposed (C1). (**B**) Final R18L tetramer-of-pentamers map with C4 symmetry imposed. Red dashed circles show the positions of a single major peak, indicating the position of the 4-fold symmetry axis. (**C**) Ab initio map of R18L co-assembled with FG peptide, C1 symmetry. (**D**) Final refined map of R18L co-assembled with FG peptide, O symmetry. Red dashed lines connect three major peaks that are oriented 90° relative to each other, consistent with octahedral symmetry.

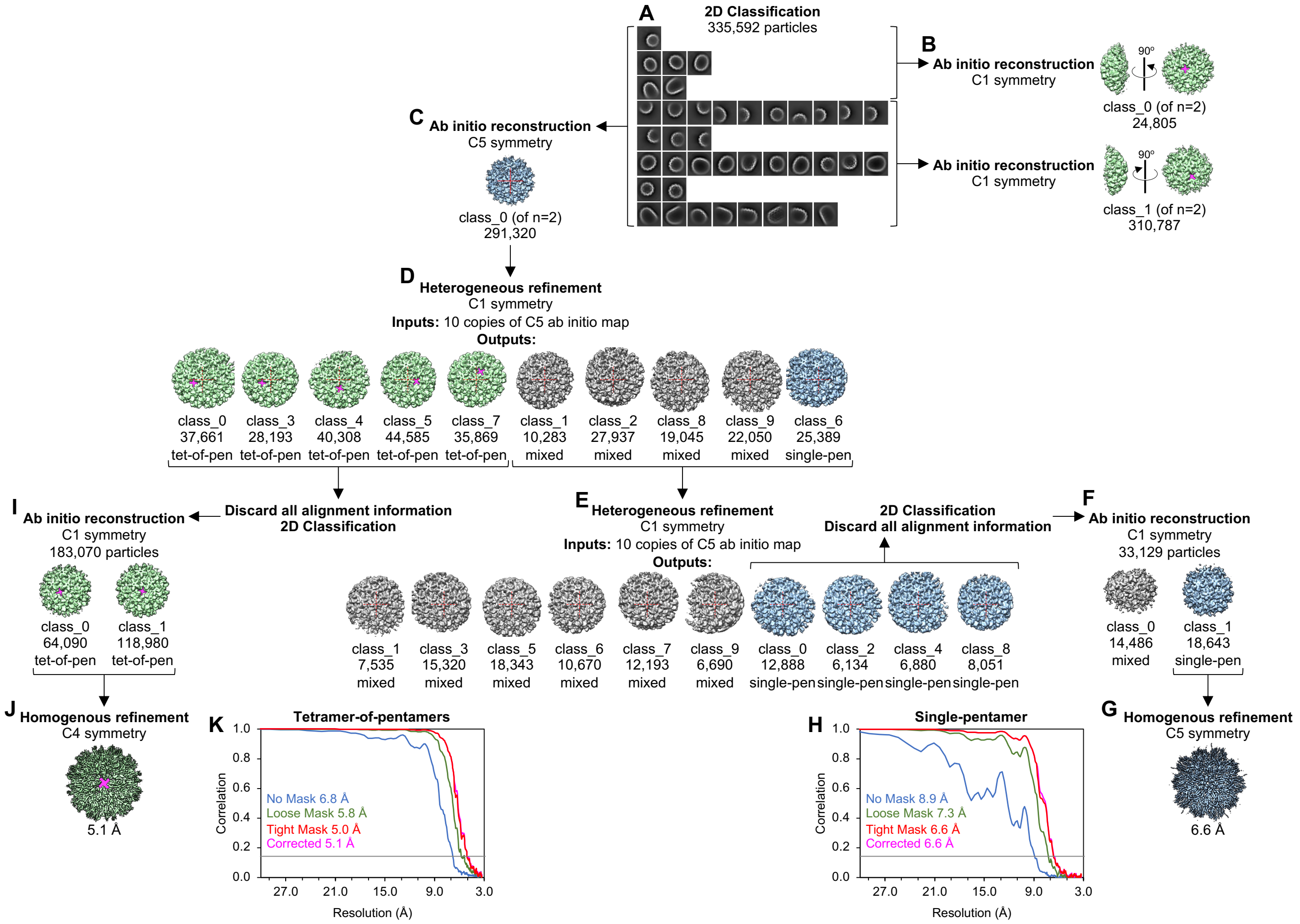

Supplementary Figure 7. Particle classification scheme to segregate the tetramer-of-pentamers (surrounded by 8 hexamers) from single-pentamers (surrounded by 5 hexamers). (**A**) Initial reference-free 2D classification. Note that each CLP will contain multiple pentamers (up to 12 of the single-pentamer type or up to 24 of the tetramer-of-pentamers type). (**B**) Ab initio maps calculated in C1 symmetry show the tetramer-of-pentamers, indicating that this is the predominant arrangement in the CLPs. Magenta marks the cross-shaped hole. (**C**) Ab initio map calculated with C5 symmetry is centered on the single-pentamer, but we expect that this initial map includes misaligned tetramers-of-pentamers. Red cross indicates the imposed 5-fold symmetry axis. (**D,E**) Two rounds of heterogeneous refinement in C1 symmetry, using 10 copies of the C5 map as input alignment template per round. Output maps recover the tetramer-of-pentamers (green), or the single-pentamer (blue), or continue to misalign the two pentamer types (gray). (**F**) For the single-pentamer particles, after discarding all previous alignment information and another round of 2D classification (clean-up), new ab initio maps (n = 2) were calculated in C1 symmetry. (**G**) Final refined map. (**H**) Fourier shell correlation curves. (**I-K**) Same as F-H, but for the tetramer-of-pentamer particles.
